# Utility of ultrasonographic examination in catheter-related infections in peritoneal dialysis: a clinical approach

**DOI:** 10.1007/s40620-023-01589-w

**Published:** 2023-03-20

**Authors:** Luca Nardelli, Antonio Scalamogna, Giuseppe Castellano

**Affiliations:** 1 Division of Nephrology, Dialysis and Kidney Transplantation, Fondazione IRCCS Ca’ Granda Ospedale Maggiore Policlinico, Via Della Commenda 15, 20122 Milan, Italy; 2 Department of Clinical Sciences and Community Health, Università Degli Studi Di Milano, Milan, Italy; 3 Division of Nephrology and Hypertension, Department of Medicine, Mayo Clinic, Rochester, MN USA

**Keywords:** Peritoneal dialysis, Exit-site infection, Tunnel infection, Peritonitis, Ultrasounds, Peritoneal catheter

## Abstract

Peritoneal dialysis- (PD) related infections continue to be a major cause of morbidity and mortality in patients on renal replacement therapy via PD. However, despite the great efforts in the prevention of PD-related infectious episodes, approximately one third of technical failures are still caused by peritonitis. Recent studies support the theory that ascribes to exit-site and tunnel infections a direct role in causing peritonitis. Hence, prompt exit site infection/tunnel infection diagnosis would allow the timely start of the most appropriate treatment, thereby decreasing the potential complications and enhancing technique survival. Ultrasound examination is a simple, rapid, non-invasive and widely available procedure for tunnel evaluation in PD catheter-related infections. In case of an exit site infection, ultrasound examination has greater sensitivity in diagnosing simultaneous tunnel infection compared to the physical exam alone. This allows distinguishing the exit site infection, which will likely respond to antibiotic therapy, from infections that are likely to be refractory to medical therapy. In case of a tunnel infection, the ultrasound allows localizing the catheter portion involved in the infectious process, thus providing significant prognostic information. In addition, ultrasound performed after two weeks of antibiotic administration allows monitoring patient response to therapy. However, there is no evidence of the usefulness of ultrasound examination as a screening tool for the early diagnosis of tunnel infections in asymptomatic PD patients.
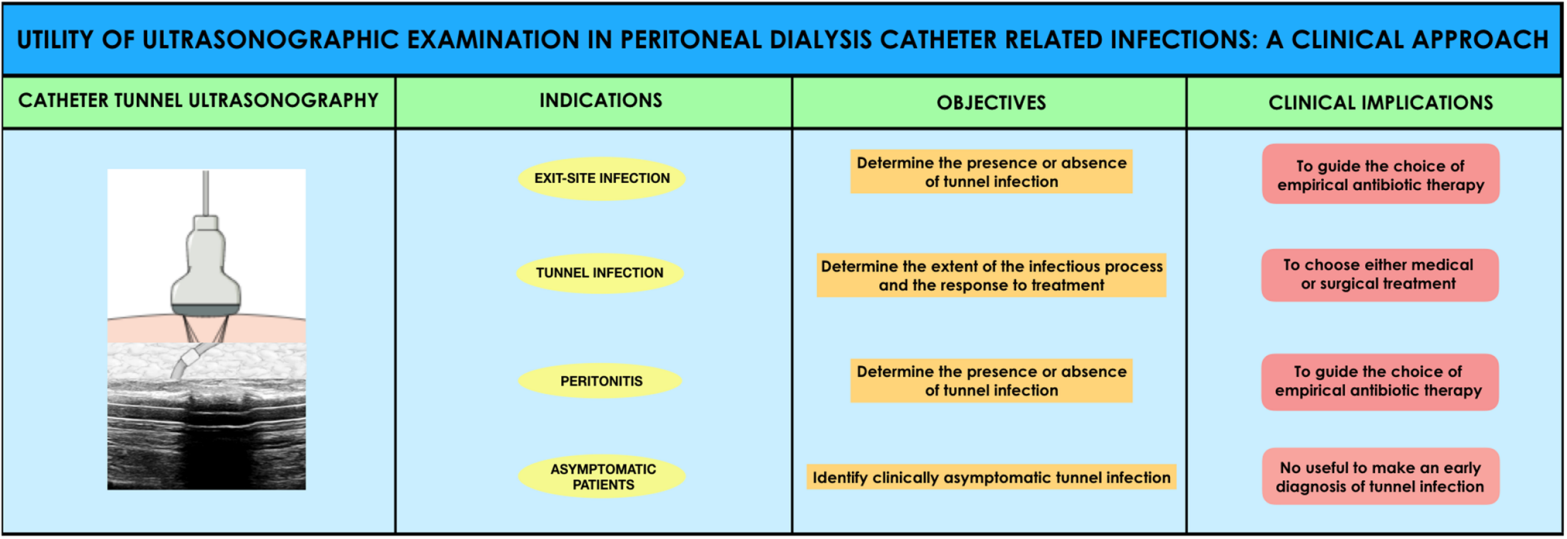

## Introduction

Infectious episodes continue to be the leading cause of morbidity and mortality in patients undergoing peritoneal dialysis (PD) [1–4]. Over the past three decades, considerable efforts have been made to prevent PD-related infections: improving connection methods, optimizing exit-site care, creating specific pathways for patient training [5–8]. Despite the adoption of these measures, approximately one third of PD failures are still secondary to peritonitis [9–12].

Recent studies support the theory that ascribes to exit-site (ESIs) and subcutaneous tunnel (TIs) infections a direct role in determining peritonitis onset [13, 14]. In particular, the ability of microorganisms to transmigrate along the tunnel from the cutaneous emergence to the peritoneal cavity has been hypothesized (periluminal route) [15, 16]. During this progression the microorganisms can colonize the Dacron of the superficial cuff and form a biofilm in this area that facilitates their proliferation [17, 18]. The creation of this layer around the superficial cuff makes these infections unresponsive to medical therapy [19, 20]. Furthermore, the bacterial colonization of the exit-site and superficial cuff would increase the probability of contamination of the patient's hands and, consequently, the passage of microorganisms into the catheter lumen during the exchange maneuvers (intraluminal route). The correct diagnosis of ESI and the timely detection of concomitant tunnel involvement would allow the rapid initiation of appropriate therapy thereby diminishing the risk of potential complications (tunnel abscess and peritonitis), while increasing the technique survival. Furthermore, early detection of the resolution of the infection would allow to adjust the duration of antibiotic therapy by minimizing the exposure of the patient to the side effects (e.g. ototoxicity, nephrotoxicity, tendon lesions, fungal peritonitis, microbial resistance). Conversely, the persistence of ultrasonographic signs attributable to the infectious process would direct the clinician to alternative therapeutic interventions (e.g. mini-surgical revision or catheter removal) [21, 22].

For this purpose, ultrasound (US) examination represents a non-invasive, relatively simple, repeatable, well tolerated and readily available method for the evaluation of the exit-site and tunnel of the peritoneal catheter [23]. Cantaluppi et al. in 1985 were the first to suggest the use of US to diagnose TIs [24]. In recent decades, the tumultuous growth of digital technology has generated fertile ground for the rapid development of ultrasonographic techniques, while the availability of equipment with high resolution capabilities has allowed an increasingly refined analysis of the diagnostic information of the echo signal, encouraging its use in several areas of modern medicine.

**Fig. 1:**
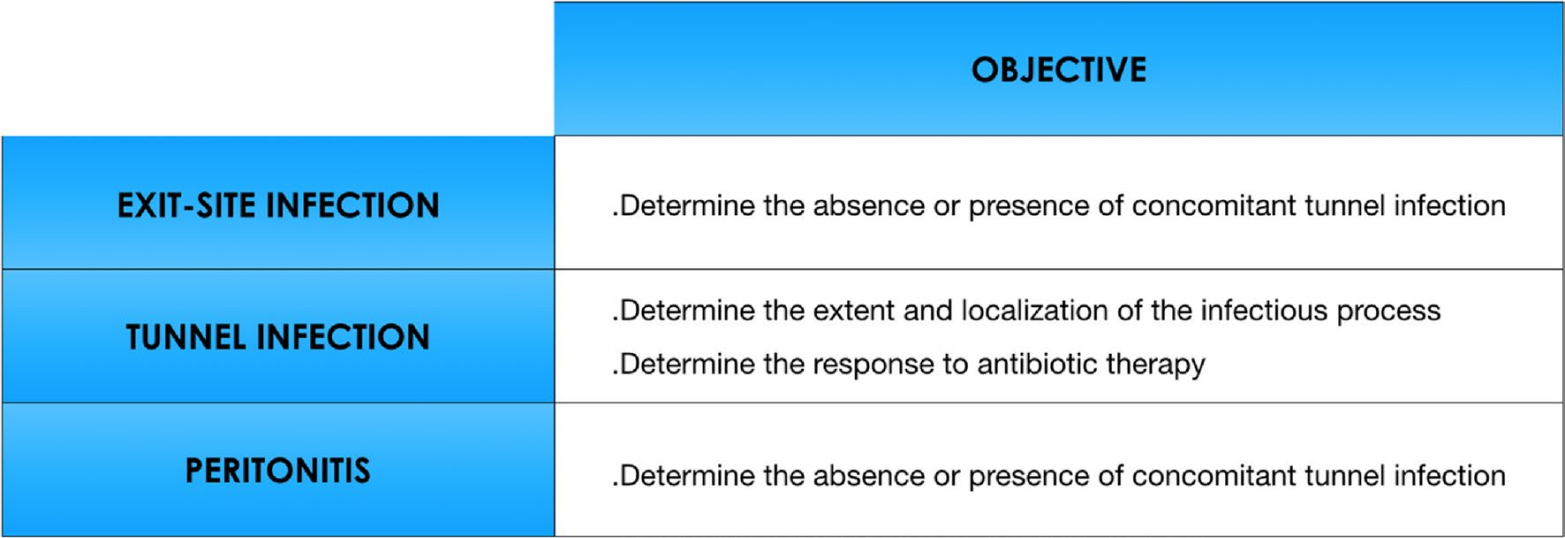
Usefulness of the ultrasonographic examination in the presence of exit-site infection, tunnel infection and peritonitis

This work, therefore, aims to analyze the indications for US examination in patients on PD with peritoneal catheter infection and to underline the clinical implications that could arise (Fig. 1).

## Ultrasonographic examination of the peritoneal dialysis catheter

US examination of the tunnel should be performed using a high frequency linear probe (7–13 MHz) and a medium–low frequency convex transducer (2.5–6 MHz) with the patient in supine decubitus. The presence of peritoneal fluid in the abdomen is optional for the examination of the extra-peritoneal portion of the catheter. Before starting the US examination, the exit-site should be carefully disinfected and covered with a transparent sterile film dressing to avoid its contamination. During this maneuver attention must be paid not to retain air bubbles between the film and the skin to avoid distortion of the signal. Using the linear probe, subcutaneous localization of the peritoneal catheter is easily accomplished by a short-axis visualization of the device at the exit-site.

**Fig. 2:**
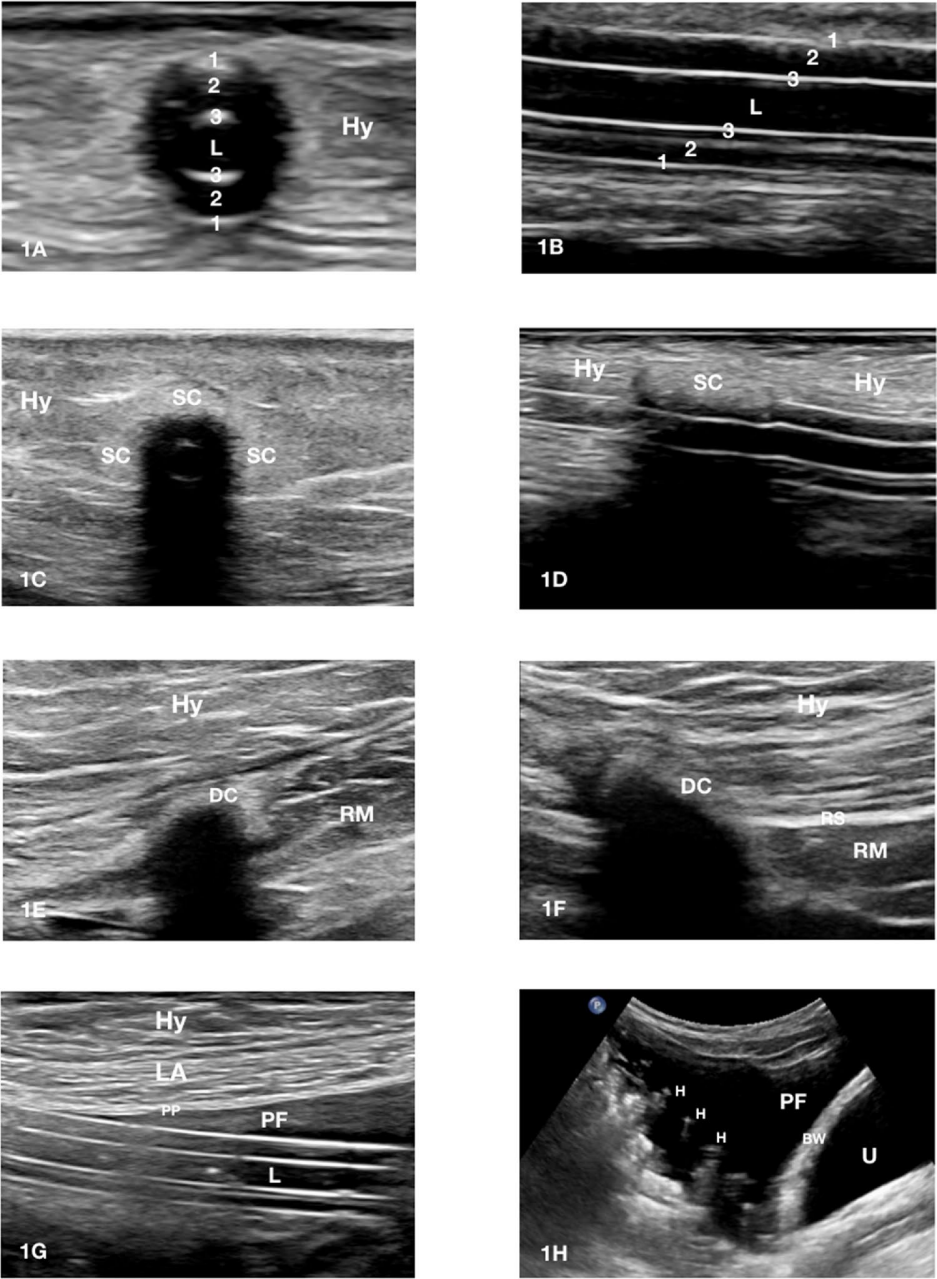
**A**–**H** normal ultrasonographic characteristics of the peritoneal catheter. **A** short-axis visualization of the catheter in the hypodermis (Hy) along the tract comprised between the exit-site and the superficial cuff. The catheter is visualized as a circular structure characterized by a trilaminar anterior wall (first thin hyperechoic rim [1], second hypoechoic layer [2], third hyperechoic rim [3]), an anechoic lumen (L) and a trilaminar posterior wall catheter (1,2,3); **B** long-axis visualization of the catheter in the subcutis along the tract comprised between the exit-site and the superficial cuff. The catheter is visualized as a “sandwich” structure characterized by a trilaminar anterior wall (1,2,3), an anechoic lumen (L) and a trilaminar posterior wall catheter (1,2,3); **C** short-axis visualization of the catheter at the level of the superficial cuff (SC). The cuff is visualized as a hyperechoic portion generating a posterior acoustic shadow; **D** long-axis visualization of the catheter at the level of the superficial cuff (SC). The cuff is visualized as a hyperechoic portion generating an acoustic posterior shadow cone; **E** short-axis visualization of the catheter at the level of the deep cuff (DC). The deep cuff possesses the same ultrasonographic features of the superficial cuff. In this case the deep cuff has been placed below the Hypodermis (Hy) within the fibers of the rectus abdominis muscle (RM). **F** long-axis visualization of the catheter at the level of the deep cuff (DC). In this case the deep cuff has been placed just above the rectus abdominis muscle (RM), (RS) = anterior rectus sheath; **G** long-axis visualization of the catheter at its passage in the peritoneal cavity. In this case the peritoneal catheter has been inserted in midline position and the deep cuff placed at the level of the linea alba (LA), as described elsewhere [48]. PP = parietal peritoneum; PL = peritoneal fluid; L = catheter lumen; **H** Visualization via convex probe (frequency range 2.5–6 MHz) of the intra-peritoneal tract of the catheter. The device is identified in the peritoneal fluid (PF) as a slightly bent curve made up of several hyperechoic spots generated by the lateral holes (H) on its distal part. In this case the tip of the catheter leans against the posterior wall of the bladder (BW) in Douglas’ pouch, U = urine

The subcutis is visualized as a superficial hypoechoic band and the catheter as a circular structure characterized by a trilaminar anterior wall (first thin hyperechoic rim, second hypoechoic layer, third hyperechoic rim), an anechoic lumen and a trilaminar posterior wall (Fig. 2A, B).

Once the catheter has been identified, it should be followed along its subcutaneous path from the exit-site until the peritoneal cavity maintaining short-axis visualization. This initial approach allows to quickly evaluate the subcutaneous course of the catheter and to promptly recognize the position of the superficial and deep cuff which varies according to the insertion technique and type of catheter. The cuffs, which are made of Dacron fibers thus poorly penetrated by ultrasounds, are displayed on the monitor as a hyperechoic portion generating a posterior acoustic shadow (Fig. 2C–F). The presence of dialysis solution inside the peritoneal cavity facilitates the recognition of the pre- and intra-peritoneal tract of the catheter (Fig. 2G). If the patient presents a thick pre-muscular adipose layer, it could be useful to evaluate this tract using a lower frequency convex probe to obtain a panoramic view of the deep elements and visualize the intracavitary course of the device (Fig. 2H). The B-Mode evaluation is then repeated by long-axis visualization of the catheter characterized by a "sandwich-like” image consisting of an anterior trilaminar wall, an anechoic central lumen and a trilaminar posterior wall, as previously described (Fig. 2B, D, G).

**Fig. 3:**
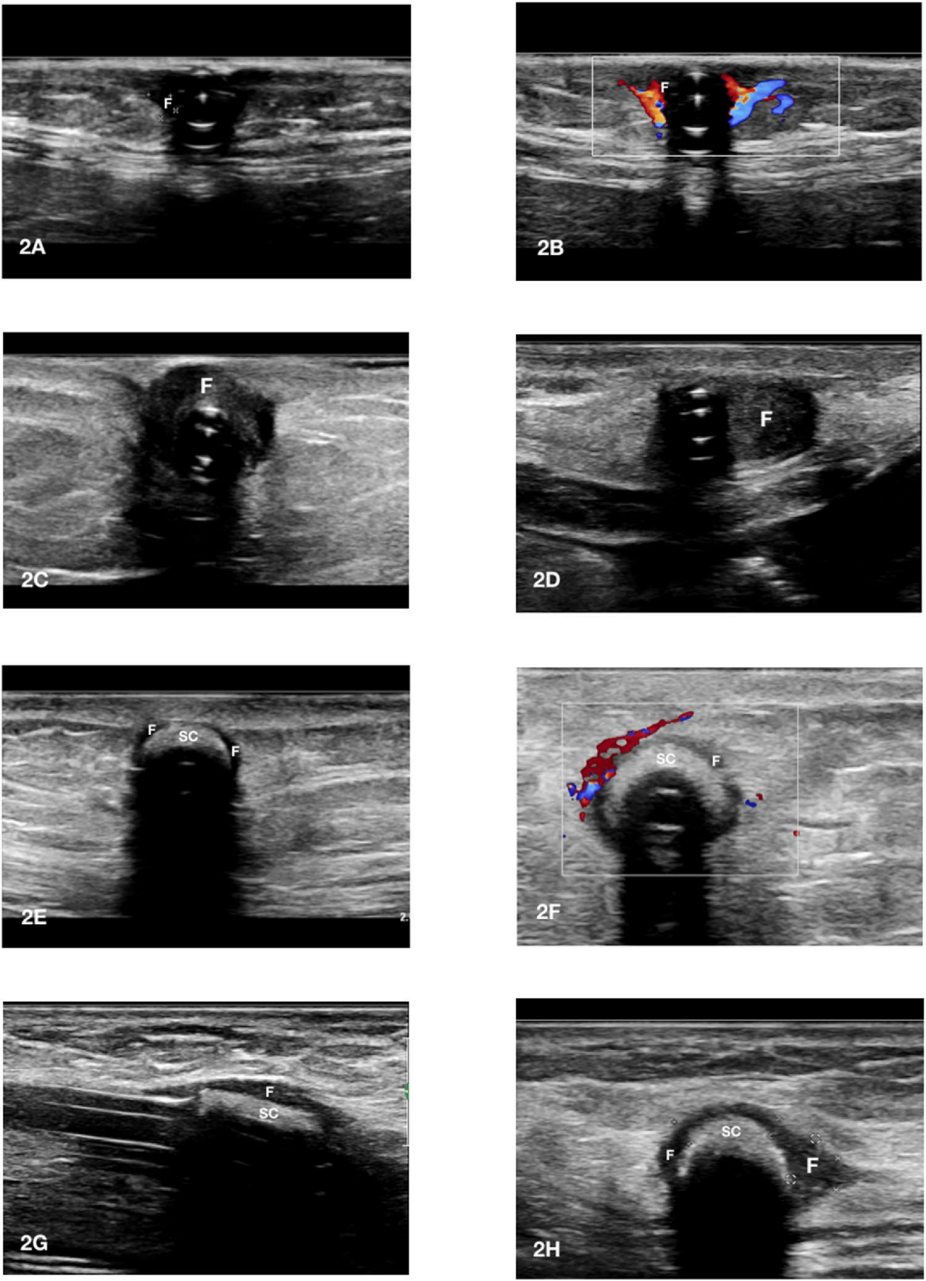
A-H tunnel infection between exit-site and superficial cuff. **A** Exit site infection with involvement of the first subcutaneous tract of the peritoneal catheter. The extent of the anechoic fluid collection (F) beside the catheter wall is comprised between 1 and 2 mm; **B** in the previous case the use of color Doppler allows to increase the diagnostic sensitivity of the ultrasonographic examination. The significant local hyperemia nearby the fluid collection (F) is suggestive for catheter infection; **C** Abscess > 5 mm in the tunnel tract upstream of the superficial cuff. The finely echoic heterogeneity of the semi-liquid collection (F) is suggestive of long-standing tunnel infection. The infectious process encircles the catheter; **D** in the previous case the infectious fluid collection (F) propagates to the left side of the catheter towards the superficial cuff; **E** tunnel infection with early involvement of the superficial cuff. The extent of the anechoic fluid collection (F) around the superficial cuff (SC) is comprised between 1 and 2 mm; **F** in the previous case the use of color Doppler allows to increase the diagnostic sensitivity of the ultrasonographic examination. The detection of local hyperemia around the superficial cuff (SC) is suggestive of cuff infection; **G** long-axis visualization confirms the localization of the fluid collection (F) around the superficial cuff (SC); **H** short axis view detects a wider abscess area (F) beside the superficial cuff (SC) that propagates towards the deep cuff

**Fig. 4:**
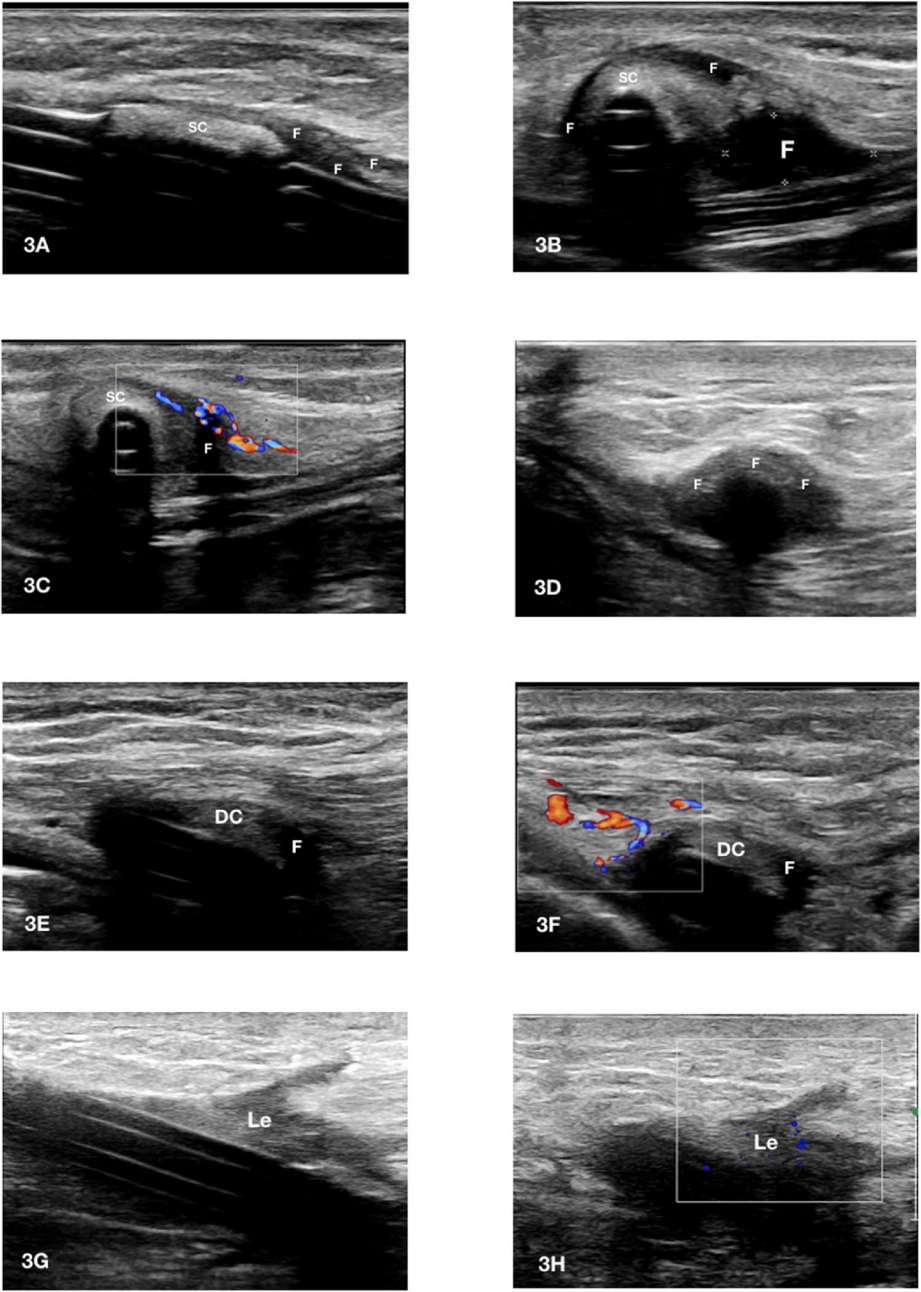
A-H tunnel infection between superficial and deep cuff. **A** This long-axis catheter view shows the propagation of the hypoechoic fluid collection (F) beyond the superficial cuff (SC); **B** in the previous case the short-axis view shows that the abscess (F) is mainly located on the left side of the catheter and from that area the infection propagates towards the deep cuff; **C** color Doppler detects the presence of active inflammation nearby the abscess (F); **D** in the catheter tract comprised between the superficial and the deep cuff a portion of the device that is frankly involved by the infection can be observed. This infectious process is likely disseminating towards the deep cuff; **E** long-axis visualization of the catheter at the level of the deep cuff (DC) shows fluid collection (F) confirming its involvement; **F** color Doppler detects the presence of active inflammation nearby the deep cuff; **G** the presence of fluid collection (Le) along the catheter tunnel in the absence of positive color Doppler signal (**H)** is suggestive of leakage or an old infectious process. In this case the diagnosis of leakage (Le) was established since the catheter had been recently inserted and the peritoneal exchanges initiated few weeks earlier

**Fig. 5:**
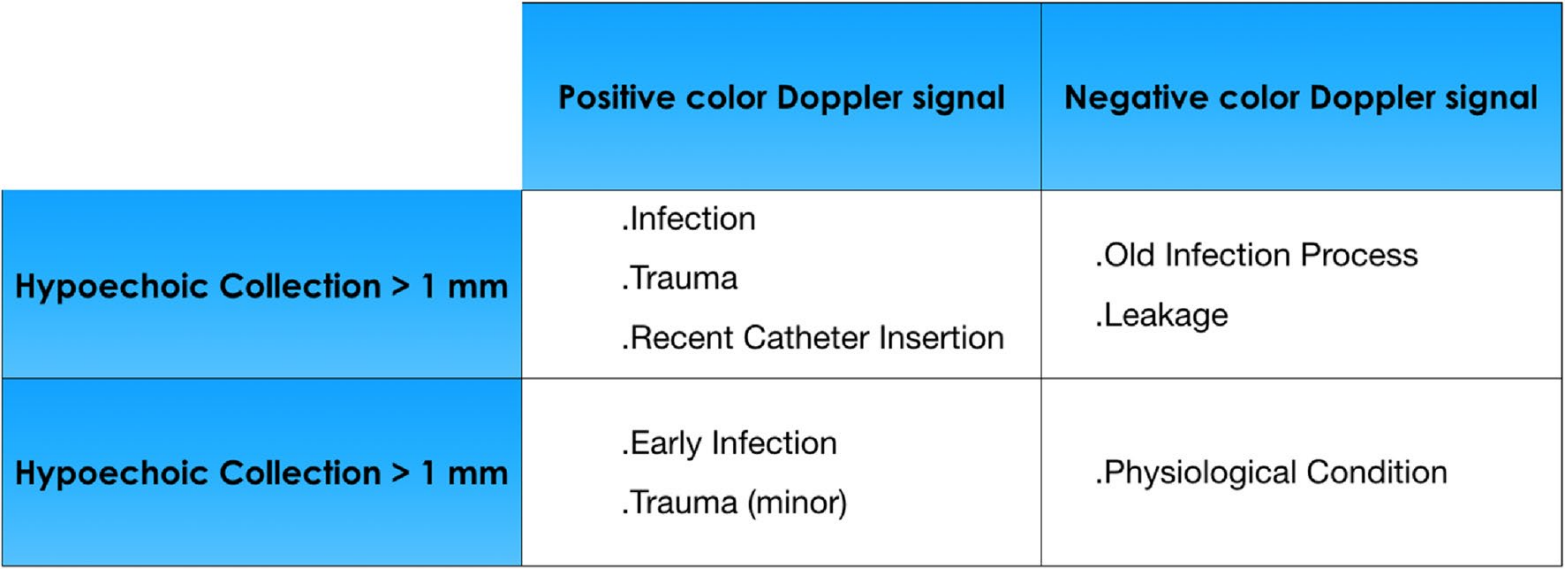
Differential diagnosis of the catheter-related event according to the extent of the hypoechoic area and the color Doppler signal

The ultrasonographic sign that suggests the existence of a TI is the presence of a hypo/anechoic collection with a diameter > 2 mm located between the catheter wall/cuff and the surrounding tissues [25–27] (Fig. 3C, D, G, H, 2A–F). More recently, a 1 mm cut-off has also been proposed [28] (Fig. 3A, B, E, F). In order to differentiate an infectious episode from leakage (Fig. 5), evaluating the region surrounding the hypo/anechoic collection using a low PRF color-Doppler module (300–800 Hz) is recommended, which in the case of an infectious process would identify an increase of color signal suggestive of local hyperemia (Figs. 3B, F–H, 4C).

## Indications for ultrasonographic examination of the tunnel

### Exit-site infection

#### Objective

**Determine the absence or presence of a concomitant tunnel infection**.

According to the guidelines of the International Society for Peritoneal Dialysis, ESI is defined by the presence of purulent discharge with or without erythema at the skin interface between epidermis and catheter [29]. Therefore, the mere presence of erythema, regardless of its extent, is not sufficient to diagnose the onset of ESI, nor is the isolation of any organism by an exit-site swab in the absence of clinical signs.

Tunnel infection, on the other hand, is defined by the presence at physical examination of inflammation (erythema, edema, pain, softening or induration) or by ultrasonographic evidence of either superficial cuff infection or hypoechoic collection along the tunnel tract [29].

In the study by Holley et al. conducted on 24 patients with a clinical diagnosis of ESI, 54% of the subjects (13 of 24) showed hypoechoic collection along the tunnel in the absence of TI signs at physical examination [30]. Similarly, Plum et al. observed that 42% of patients affected by ESI (18 of 43) showed the presence of a hypoechogenic layer along the subcutaneous tunnel in the absence of erythema, softening or induration [25]. These data were confirmed also by Korzets [26] and Kwan [28] who reported that approximately 50% of patients with a diagnosis of ESI but without obvious signs of TI showed positive tunnel US. Although Vychytil et al. did not report a greater ability of US examination to identify TI as compared to clinical criteria, there is enough evidence to state that in the presence of an established ESI, ultrasonographic examination possesses greater sensitivity in diagnosing asymptomatic infections of the catheter portion comprised between the superficial and deep cuff [31].

The simultaneous involvement of the tunnel during ESI represents a significant prognostic factor. Approximately 50% of ESIs associated with TIs are sustained by Staphylococcus aureus [25, 30–32] which leads to secondary peritonitis in more than 50% of patients, thus requiring removal of the catheter in most cases [25, 28, 30–32]. On the contrary, no catheters were lost in patients with ESI who had no tunnel involvement.

Thus, in the setting of ESIs, ultrasonographic examination of the tunnel has proved to be as specific as the clinical parameters, but with considerably greater sensitivity, especially in case of TIs involving the tunnel portion comprised between the superficial and deep cuff. Furthermore, it allows to distinguish ESIs that are likely to be successfully treated with oral antibiotic therapy alone from those that may require more intensive treatment (e.g. intravenous antibiotic therapy, removal of the superficial cuff or catheter) [25, 30, 31, 33].

#### Clinical implications

**Fig. 6:**
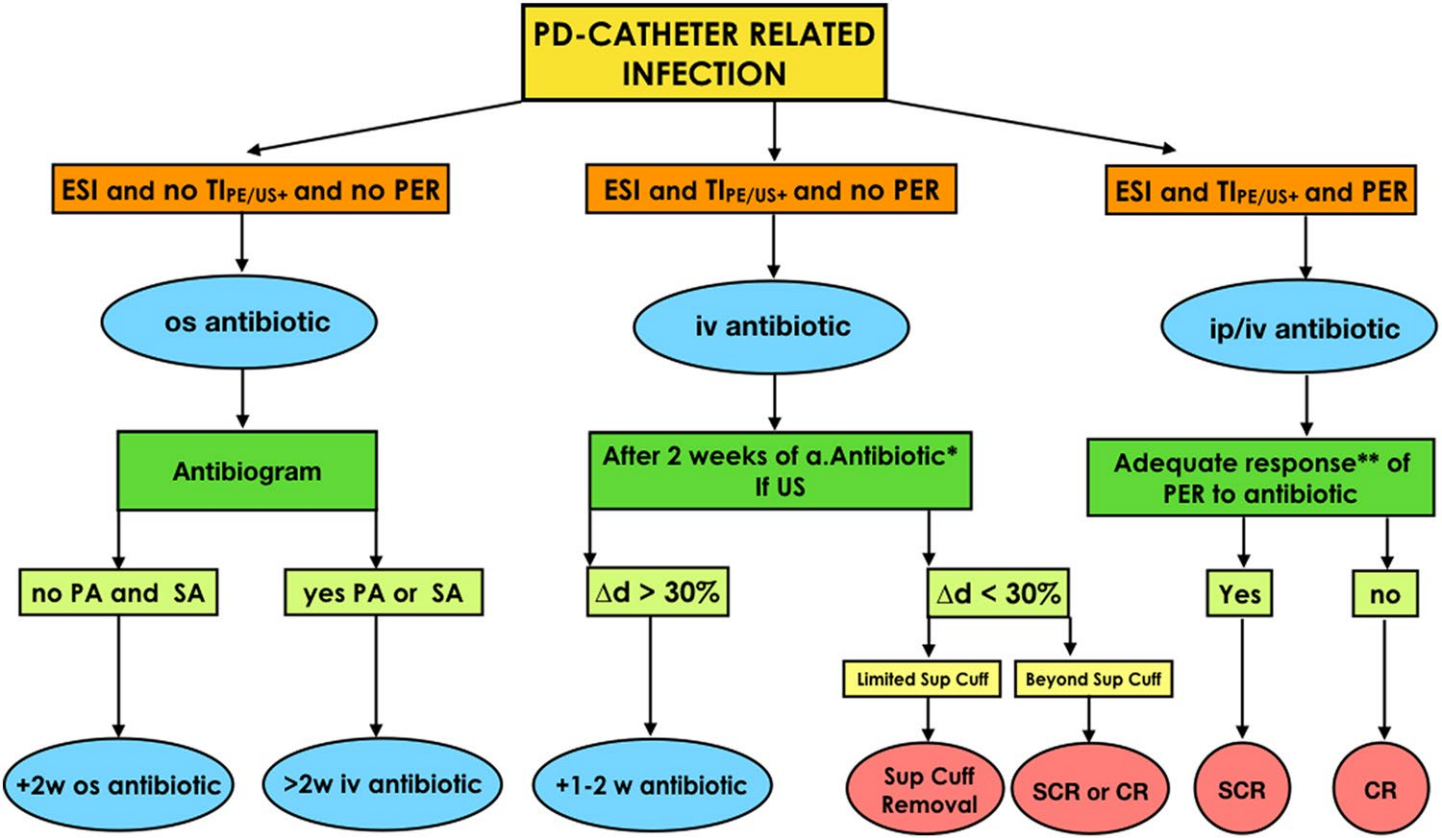
Flowchart of exit-site infection (ESI), tunnel infection (TI) and peritonitis (PER) management according to an ultrasonographic-integrated approach. *CR* catheter removal, *iv* intravenous, *ip* intraperitoneal, *os* oral, *PA* Pseudomonas aeruginosa, *PD* peritoneal dialysis, *SA Staphylococcus aureus*, *SCR* simultaneous insertion and removal of the catheter, *TI*_*PE/US*+_ presence of positive physical or ultrasonographic exam for tunnel infection, *Sup cuff* superficial cuff, *US* ultrasonographic exam, *w* weeks, *Δd* difference of the hypoechoic area diameter after 2 weeks of antibiotic therapy; after 2 weeks of a.Antibiotic * = antibiotic therapy based on antibiogram; adequate response** = dialysis effluent white cell count was < 100/μL (after a dwell time of at least 2 h) for 4 days in a row [33]

Whenever an episode of ESI is diagnosed, performing US of the tunnel is recommended in order to obtain initial diagnostic and prognostic information that can guide the choice of empirical therapy. In the case of ESI without tunnel involvement and a history of methicillin-resistant *S. aureus* and/or Pseudomonas infections, it is reasonable to start empirical oral antibiotic therapy against *S. aureus*, which hypothetically should last at least 2 weeks. On the other hand, in case of ESI with concomitant TI, prescription of intravenous (IV) therapy is suggested, which hypothetically should last at least 3 weeks [29] (Fig. 6). Type of antibiotic and duration of therapy must then be adjusted according to the results of the culture and the clinical response to therapy.

### Tunnel infection

#### Objective

**Determine the extent and localization of the infectious process.**

Over time, in an effort to minimize mechanical and infectious complications, several types of peritoneal catheters have been designed which differ from the original Tenckhoff catheter [34] with regard to tip conformation (straight/coiled), type of subcutaneous portion (straight/pre-curved), length of the intraperitoneal portion (15 or 8 cm) [35], presence of extensions (exit-site at the upper abdominal or pre-sternal exit-site) or additional tools to prevent the dislocation of the intraperitoneal tract (Toronto/Ash and Janle catheter/Di Paolo) [36–38]. However, the unifying characteristic is the presence of one or more Dacron cuffs provided to facilitate the anchoring of the catheter to the surrounding tissue. It is possible to divide the PD catheter into different regions using the cuffs as reference points. In particular, the single cuff catheter can be split into two parts (the portion upstream of the cuff and the portion downstream), while the double cuff catheter can be divided into three parts (the portion between the exit-site and the superficial cuff, the tract between the two cuffs and the part between the deep cuff and the tip of the catheter).

In this regard, evidence in the literature suggests that the specific localization of the abscess as assessed by US possesses a significant prognostic value in the setting of PD catheter-related infections. Vychytil et al. divided the infectious episodes into three categories based on US: isolated exit-site infection (ESI without signs of TI), superficial tunnel infection (absence of cuff involvement) and deep tunnel infection (ultrasonographic signs of deep cuff involvement). The Authors observed that all cases of exit-site and superficial tunnel infections were efficaciously treated with antibiotic treatment, while 40% of the deep tunnel infections were refractory to medical therapy thus necessitating catheter removal [31]. In patients with double cuff catheter, recognition of deep cuff infection was strongly associated (> 90% of the cases) with infection recurrence [28]. Similarly, Plum et al. observed that the presence of a positive US at the segment of the catheter between the two cuffs predicted the occurrence of a secondary peritonitis episode in 62.5% of cases [25]. Data from the literature suggest, therefore, that tunnel infections with cuff involvement are unlikely to respond to antibiotic therapy and in most cases will require catheter removal.

#### Objective

**Follow-up during treatment**.

Once a catheter-related infection has been diagnosed and antibiotic therapy initiated, the need to monitor treatment response arises so as to determine the duration of the medical therapy and evaluate its effectiveness.

Domico was the first to describe the negative prognostic value of the persistence of US positivity. In this case series, 80% of patients who after 4 weeks of appropriate antibiotic therapy continued to show signs of infection at US required catheter removal [32]. Analyzing the outcome of patients with deep tunnel infections, Vychytil et al. investigated whether the extent and variation of the pericatheter collection could provide significant predictive information [31]. In particular, the authors did not find a statistically significant difference in the extent of liquid collection before the start of antibiotic therapy between subjects who experienced catheter loss compared to those who recovered with medical therapy (5.49 ± 0.58 mm vs 7.02 ± 0.7 mm, respectively). However, 1 week after starting antibiotic therapy, the group of patients who eventually did not require catheter removal showed a decrease in liquid collection extent (6.48 ± 1.05 mm), which became even more significant after 2 weeks of treatment (3.75 ± 1.04 mm), while in the group of patients requiring catheter removal the liquid collection remained essentially unchanged after one (5.34 ± 0.49 mm) and 2 weeks (5.06 ± 0.38 mm) of treatment. In addition, 85% of cases of deep tunnel infections that responded to medical therapy showed an extension decrease of the anechoic area greater than 30%, unlike all cases of TIs that required catheter removal [31]. Similarly, Kwan et al. reported a significantly worse outcome (73% recurrence of infectious episodes and 27% of catheter removal at three months) in patients who after two weeks of antibiotic therapy showed fluid collection increase greater than 1 mm [28].

Therefore, ultrasound in association with clinical-laboratory data proved to be a useful tool for monitoring response to medical therapy in patients with TIs.

#### Clinical implications

Making use of US in order to early recognize TIs with involvement of the deep cuff or the tunnel segment between the cuffs is recommended. These conditions represent a negative prognostic factor for short-term recurrence indicating the need for more aggressive medical therapy [29].

Subsequently, the effectiveness of the treatment should be based on US performed two weeks after the start of adequate antibiotic therapy adjusted according the antibiogram. If the decrease in liquid collection is greater than 30% of the initial extent, it is likely that the infectious process will resolve by medical therapy. In this case a surgical approach should be avoided, and antibiotic prolonged for at least another week. At the end of the therapy, it would be useful to repeat US to confirm the complete disappearance of the peri-catheter hypoechoic zone and color Doppler signal.

On the other hand, if the total accumulation does not decrease by at least 30% after 2 weeks of appropriate antibiotic therapy, proceeding with the removal of the superficial cuff and prolonging antibiotic therapy is recommended if the US does not detect an extension of the infection beyond the superficial cuff [21, 39, 40]. Conversely, if the infection has spread to the deep cuff or to the portion of the tunnel between the two cuffs, removal of the peritoneal catheter is indicated (Fig. 6). In the absence of active secondary peritonitis, simultaneous removal and insertion of a new PD catheter should be pursued if the expertise of the center allows to perform the procedure safely [33, 41–43] (Fig. 6).

### Peritonitis

#### Objective

**Determine the absence or presence of a concomitant tunnel infection**.

Recently, thanks to the acquisition of further data concerning peritonitis in patients on PD, specific clinical entities have been described [44, 45], such as “relapsing peritonitis” (episode of peritonitis which occurs within 4 weeks of the conclusion of therapy undertaken for the treatment of a previous peritonitis sustained by the same microorganism) and “repeated peritonitis” (episode of peritonitis that occurs more than 4 weeks after the conclusion of therapy undertaken for the treatment of a previous peritonitis sustained by the same microorganism) [46]. However, the risk factors for relapsing or recurring peritonitis after a first episode of peritonitis have not been identified with certainty. In the study by Karahan et al. [27], US of the tunnel was performed in conjunction with any infectious event including peritonitis. The authors observed that even in the absence of any clinical signs or symptoms, simultaneous involvement of the cuffs and/or tunnel documented by US was present in approximately 55% of patients. Similarly, Korzets et al. identified by US simultaneous involvement of the tunnel in 62% of patients with a clinical diagnosis of peritonitis [26]. Notably, 25% of these patients showed peritonitis compared with no patients with negative US.

#### Clinical implications

Despite the limited evidence, it could be useful to perform US upon presentation of any peritonitis episode in order to identify cases with concomitant involvement of the tunnel which, being less likely to respond to medical therapy, may require more aggressive and longer-lasting empirical antibiotic treatment [46] (Fig. 6).

### Screening in asymptomatic patients

#### Objectives

**Identify clinically asymptomatic tunnel infections**.

Based on the assumption that US examination possesses greater sensitivity than physical examination in the diagnosis of TIs, the utility of performing tunnel ultrasonographic evaluation in every asymptomatic patient at defined intervals has been investigated. Plum et al. performed 548 US examinations on 62 patients at each outpatient visit (4–8-week intervals) and identified only 3 ultrasound-documentable cases of TI in the absence of any symptoms related to a catheter infection [25].

The limited usefulness of using US of the tunnel as a screening tool was confirmed by Vychytil et al. who performed 199 US examinations of the tunnel in asymptomatic patients and did not observe any results clearly suggestive of TI [47].

### Clinical implications

There are no data in favor of performing US examination of the catheter tunnel at predefined intervals in asymptomatic patients on PD in order to make a very early diagnosis of any possible infectious processes (Fig. 6).

## Future developments

Although several experiences are currently available regarding the use of US in catheter-related infections, it is necessary to consolidate the evidence with well-planned studies.

Further data are required to more clearly establish the prognostic value of the localization of the catheter infection. In fact, the current definition of TI does not differentiate infections limited to the superficial cuff from infectious events that extend to the tract between the two cuffs and/or to the deep cuff. In case of antibiotic failure, a TI classification based on US results could identify the episodes that require a mini-invasive surgical approach (e.g. cuff shaving, removal of the superficial cuff, partial reimplantation of the catheter) from those that require removal of the catheter altogether.

Further data are still needed to verify the role of US in predicting the response to antibiotic therapy. For this purpose, US of the tunnel should always be repeated two weeks after initiating antibiotic therapy. Furthermore, the usefulness of ultrasonographic diagnosis in the follow-up of peritonitis episode remains to be proven. In particular, the persistence at US of an accumulation along the tunnel or beside the cuffs could allow the identification of subjects at greater risk of relapsing or repeated peritonitis.

Similarly, the role of color/power Doppler in the setting of catheter-related infections is yet to be defined. From a pathophysiological standpoint, at the beginning of an infection an increase in local vascularity occurs, that precedes the onset of edema or collection. Therefore, the detection of color Doppler signal in the absence of hypoechoic collection could anticipate the diagnosis of infection (Fig. 5). During antibiotic therapy there should be a reduction in hypoechoic collection concomitant with a reduction in local hypervascularization. However, apart from some reports concerning personal clinical experience, no data are available regarding the diagnostic and prognostic value of these techniques.

Vychytil et al. reported that 15 out of 199 US screening tests were questionable [47]. These episodes were subsequently identified as being negative on the basis of the absence of development of clinical symptoms/signs and lack of change in the ultrasound picture at subsequent examinations. The use of color Doppler could promptly guide the differential diagnosis of such cases [Fig. 5]. Speculatively, it cannot be excluded that the creation of a specific “severity scale” based on the color Doppler aspect may allow the acquisition of notions that can be integrated with the B-mode technique in order to obtain more accurate prognostic information.

Quantification of the Doppler signal, which also depends on the subjective ultrasound setup, remains a problem to be solved. Finally, color Doppler could be useful in the early phase of infection in the presence of a hypoechoic pericatheter area less than 2 mm (Fig. 5).

## Conclusion

Ultrasonographic examination of the tunnel in patients on PD represents a useful tool in all cases of catheter-related infection. During an episode of ESI, US allows to diagnose a concomitant tunnel infection with greater sensitivity than by clinical parameters alone. In addition, US can help to more accurately distinguish ESIs that will likely resolve with oral antibiotic therapy from those that may require a more aggressive therapeutic approach (intravenous antibiotic therapy, surgical revision of the tunnel, removal of the catheter). In case of TI, US initially allows to accurately locate the segment of the catheter affected by the infectious process, thus helping the clinician take into consideration the surgical approach when the ultrasound signs of cuff infection remain unchanged over time. Subsequently, the repetition of the US examination 2 weeks after the start of the antibiotic allows to monitor treatment response, supporting the decision to either extend the antibiotic therapy or to proceed with a surgical intervention. On the other hand, in episodes of peritonitis, although the evidence regarding the utility of the tunnel is still limited, the detection of tunnel involvement represents an important piece of prognostic information in guiding the clinician to choose the best therapeutic approach. However, there is no evidence to support the use of tunnel US as a screening tool for the early detection of TIs in asymptomatic patients. Although the usefulness of US in the diagnosis and management of infections related to the peritoneal catheter is indisputable, it is necessary to confirm the available evidence with rigorously planned studies conducted on larger populations.

## Data Availability

The datasets generated during and/or analysed during the current study are available from the corresponding author on reasonable request.
